# Simultaneous PET-MRI imaging of cerebral blood flow and glucose metabolism in the symptomatic unilateral internal carotid artery/middle cerebral artery steno-occlusive disease

**DOI:** 10.1007/s00259-019-04551-w

**Published:** 2019-11-06

**Authors:** Bixiao Cui, Tianhao Zhang, Yan Ma, Zhongwei Chen, Jie Ma, Lei Ma, Liqun Jiao, Yun Zhou, Baoci Shan, Jie Lu

**Affiliations:** 1 Department of Nuclear Medicine, Xuanwu Hospital Capital Medical University, Beijing, China; 2 Beijing Engineering Research Center of Radiographic Techniques and Equipment, Institute of High Energy Physics, Chinese Academy of Sciences, Beijing, China; 3 School of Nuclear Science and Technology, University of Chinese Academy of Sciences, Beijing, China; 4 Department of Neurosurgery, Xuanwu Hospital Capital Medical University, Beijing, China; 5 GE Healthcare, Beijing, China; 6 Mallinckrodt Institute of Radiology, Washington University in St. Louis School of Medicine, St. Louis, MO USA; 7 CAS Center for Excellence in Brain Science and Intelligence Technology, Shanghai, China; 8 Department of Radiology, Xuanwu Hospital Capital Medical University, Beijing, China

**Keywords:** PET/MR, Glucose metabolism, Cerebral blood flow, Superficial temporal artery-middle cerebral artery bypass, Ischaemic cerebrovascular disease

## Abstract

**Purpose:**

Cerebral blood flow (CBF) and glucose metabolism are important and significant factors in ischaemic cerebrovascular disease. The objective of this study was to use quantitative hybrid PET/MR to evaluate the effects of surgery treatment on the symptomatic unilateral internal carotid artery/middle cerebral artery steno-occlusive disease.

**Methods:**

Fifteen patients diagnosed with ischaemic cerebrovascular disease were evaluated using a hybrid TOF PET/MR system (Signa, GE Healthcare). The CBF value measured by arterial spin labelling (ASL) and the standardized uptake value ratio (SUVR) measured by ^18^F-FDG PET were obtained, except for the infarct area and its contralateral side, before and after bypass surgery. The asymmetry index (AI) was calculated from the CBF and SUVR of the ipsilateral and contralateral cerebral hemispheres, respectively. The ΔCBF and ΔSUVR were calculated as the percent changes of CBF and SUVR between before and after surgery, and paired *t* tests were used to determine whether a significant change occurred. Spearman’s rank correlation was also used to compare CBF with glucose metabolism in the same region.

**Results:**

The analysis primarily revealed that after bypass surgery, a statistically significant increase occurred in the CBF on the affected side (*P* < 0.01). The postprocedural SUVR was not significantly higher than the preprocedural SUVR (*P* > 0.05). However, the postprocedural AI values for CBF and SUVR were significantly lower after surgery than before surgery (*P* < 0.01). A significant correlation was found between the AI values for preoperative CBF and SUVR on the ipsilateral hemisphere (*P* < 0.01).

**Conclusions:**

The present study demonstrates that a combination of ASL and ^18^F-FDG PET could be used to simultaneously analyse changes in patients’ cerebral haemodynamic patterns and metabolism between before and after superficial temporal artery-middle cerebral artery (STA-MCA) bypass surgery. This therefore represents an essential tool for the evaluation of critical haemodynamic and metabolic status in patients with symptomatic unilateral ischaemic cerebrovascular disease.

## Introduction

Ischaemic cerebrovascular disease is a chronic, occlusive cerebrovascular disease of unknown aetiology that is characterized by progressive steno-occlusive changes at the terminal portion of the internal carotid artery (ICA) or middle cerebral artery (MCA). Symptomatic patients are treated by various methods, including aggressive medical management, intracranial angioplasty, stenting, and cerebrovascular bypass surgery [1–3]. The quantification of cerebral blood flow (CBF) is important and meaningful in ischaemic stroke [4]. Previous studies reported that while the hypoperfusion area (**<** 25 mL/100 g/min) was not primarily damaged by a reduction in blood supply, affected patients should undergo bypass surgery to improve CBF [5, 6]. Positron emission tomography (PET) imaging with ^15^O-H_2_O is considered the gold standard for measuring CBF [7, 8]. However, the standard protocol is not widely used in the clinic due to its invasiveness. An alternative option, arterial spin labelling (ASL), is a promising non-invasive method that can be used to measure CBF that is also convenient to apply [9, 10]. Previous researchers who obtained CBF values using both ^15^O-H_2_O PET and ASL-MRI found that there was a significant correlation between the two methods when used in healthy subjects under identical physiological conditions [11, 12]. In addition, decreased CBF (< 35.0 mL/100 g/min) was observed on the affected side, indicating chronic hypoperfusion [13, 14].

However, CBF alone is not sufficient to predict tissue fate. Normally, because glucose is the primary source of energy for brain cells, glucose metabolism also plays an important role in characterizing functional metabolism [15, 16]. ^18^Fluoro-2-deoxy-D-glucose (^18^F-FDG) is widely used in different diseases as a common tracer and in the measurement of brain glucose metabolism. Recently, there has been tremendous interest in evaluating the potential of ^18^F-FDG in cerebral ischaemia [17–20]. Deng et al. also observed that ^18^F-FDG uptake was higher in the cerebral infarction area among drug treatment groups in a rat model of cerebral ischaemia reperfusion injury evaluated after 7 days [18]. Many previous studies have investigated perfusion and glucose metabolism using separate PET and MR procedures or integrated PET/MR in patients with cancer, Alzheimer’s disease, epilepsy, and other disorders [21–23]. MR images and PET images can be obtained at different time points and fused for clinical and research purposes. However, various physiological processes may change between these imaging sessions. The optimal approach is to simultaneously acquire data using different modalities. Hybrid PET/MR solves this problem by offering complementary information obtained via conventional MR imaging, which has high spatial resolution and tissue contrast, and PET, which provides physiological information.

Superficial temporal artery MCA (STA-MCA) bypass is an effective method of surgical revascularization used in the treatment of ischaemic cerebral artery disease. The most important goal of surgical revascularization is to improve CBF as assessed by imaging [24]. Effective revascularization strategies improve haemodynamic parameters and reduce stroke occurrence [13, 25]. Cerebral FDG metabolism is also one of the factors used to evaluate cerebral metabolism [26]. ^18^F-FDG PET imaging shows promise as an indicator of the effectiveness of therapies in cerebral ischaemia [19]. Yoshida, K. et al. combined measurements of CBF and cerebral glucose metabolism to evaluate cognitive changes after carotid endarterectomy [27], and Yu Z et al. used ^18^F-FDG PET/CT to evaluated 7 patients who underwent STA-MCA bypass surgery [28]. However, few studies have focused on combining measurements of CBF and ^18^F-FDG uptake, especially using an integrated simultaneous PET/MR scanner, to evaluate patients after STA-MCA bypass surgery.

In this study, the effects of surgical treatment on symptomatic unilateral ICA/MCA steno-occlusive disease were evaluated using quantitative hybrid PET/MR. We first investigated the potential role of a simultaneous PET/MR system in patients with chronic and symptomatically severe steno-occlusive disease of the ICA or MCA in the same functional and physiological states. Second, we analysed changes in cerebral haemodynamic patterns and metabolism that occurred in patients who were offered superficial temporal artery (STA)–MCA bypass surgery as well as changes in imaging findings on follow-up scans obtained at 7 days after surgery. Third, we assessed the correlation between CBF measured by ASL and metabolism assessed by ^18^F-FDG in patients with symptomatic unilateral ischaemic cerebrovascular disease.

## Materials and methods

### Patients

In all, 22 patients satisfied the inclusion criteria, but only 15 (13 male and 2 female subjects; weight, 73.13 ± 10.29 kg; height, 167.00 ± 8.24 cm) with a mean age of 47.33 years underwent follow-up PET/MR (Table 1). The main inclusion criteria were (1) a confirmed diagnosis of ICA or MCA occlusive disease based on digital subtraction angiography [29]; (2) a history of transient ischaemic attacks or complete stroke involving the relevant ICA or MCA territory and treatment with ineffective medication [4, 30]; and (3) completed PET/MR scanning within 1 month before surgery and had confirmed vascular connection success based on digital subtraction angiography after surgery. The exclusion criteria included any contraindication for MRI and artefacts on MRI. Fifteen out of the 22 patients underwent a repeat follow-up PET/MR scan 7 days after STA-MCA anastomosis. The subjects provided written informed consent for the study protocols, which were approved by the ethics committee of Xuanwu Hospital and conducted in accordance with the Declaration of Helsinki.

**Table 1:** Summary and basic characteristics of the patient population

Mean ± SD of patient age (years)	47.33 ± 12.65
Sex (M/F)	12:03
Mean ± SD of blood glucose (mmol/L)	
Preprocedural	5.85 ± 0.85
Postprocedural	5.83 ± 0.73
Mean ± SD of injection (MBq)	
Preprocedural	295.26 ± 51.06
Postprocedural	277.87 ± 40.70

### PET/MR acquisition

All images were acquired on a hybrid TOF PET/MR system (Signa, GE Healthcare). TOF PET and MR images were simultaneously acquired in 19-channel head and neck union coil. Patients were placed in a supine position and instructed to remain calm with their eyes closed. Each patient was instructed to fast for at least 6 h to reach a serum glucose level lower than 8 mmol/L and received a manual, intravenous injection of ^18^F-FDG (3.7 MBq/kg). Fifty minutes after this injection, the patients were placed in the PET/MR scanner. ^18^F-FDG PET images were acquired for 10 min. The PET data were subjected to attenuation correction, scatter correction, random correction, decay correction, and dead-time correction [31]. Attenuation correction was performed based on MR images, and the default attenuation correction sequence (Dixon MR sequences) was automatically prescribed and acquired as follows: LAVA-Flex (GE Healthcare) axial acquisition, repetition time (TR) = 4 ms, echo time (TE) = 1.7 ms, slice thickness = 5.2 mm with 2.6-mm overlap, 120 slices, pixel size = 1.95 × 2.93 mm, and acquisition time = 18 s. Corrected PET data were obtained using a time-of-flight, point spread function, ordered subset expectation maximization (TOF-PSF-OSEM) algorithm with 8 iterations and 32 subsets, and a 3-mm cut-off filter. The image matrix was 192 × 192, the field of view was 35 × 35 cm^2^, and the pixel size was 1.82 × 1.82 × 2.78 mm^3^.

PET and MR imaging data were simultaneously acquired. The main MR sequences included T2 fluid-attenuated inversion recovery (FLAIR) and 3D ASL. For FLAIR, the following parameters were applied: T2-FLAIR with 32 slices, TR = 11000 ms, TE = 144 ms, the voxel size = 0.47 × 0.47 × 4.00 mm^3^, and scan time = 2:56 min. For 3D ASL, the following parameters were applied: 36 slices, postlabelling delay = 2.5 s, TR = 5335 ms, TE = 10.7 ms, the voxel size = 1.88 × 1.88 × 4.00 mm^3^, and scan time = 5:10 min [9].

### Data analysis

T2-FLAIR images were applied to measure the infarction area in each patient. Two experienced radiologists individually measured the infarction areas using a T2-FLAIR image in blind mode. The average values calculated by the two radiologists were used to define the infarction volume in each patient.

All images were preprocessed using SPM8 (Wellcome Department of Clinical Neurology, London, UK). ^18^F-FDG PET and ASL images were first spatially normalized to the MNI (Montreal Neurological Institute) space with a 3 × 3 × 3 mm^3^ resolution using the affine transformation and subsequent nonlinear warping. Then, the ^18^F-FDG PET images were transformed into maps representing the SUVR, which was defined as the tissue concentration of radioactivity (kBq/mL) in each voxel normalized to the mean activity concentration in a reference region. Since the infarction influences the glucose metabolism of the contralateral cerebellum [32, 33], only the ipsilateral cerebellum was used as the reference region. Finally, all images were smoothed using an isotropic Gaussian kernel at full width at half maximum (FWHM) of 8 mm in all directions. CBF and SUVR values were obtained excluding the infarct area and its contralateral side.

### CBF measurements

The CBF was computed using the following equation [34]:

$$ \mathrm{CBF}=6000\frac{\lambda \left(1-\exp \left(-\frac{T_{SAT}}{T{1}_{GM}}\right)\right)\exp \left(\frac{w}{T{1}_B}\right)}{2T{1}_B\left(1-\exp \left(-\frac{\tau }{T{1}_B}\right)\right)\varepsilon}\left(\frac{ASL\mathrm{diff}}{\left(45.25\mathrm{nex}\right)\mathrm{PDref}}\right) $$

where the postlabelling delay was *w*=2.5 s, the labelling time was *τ*=1.5 s, the partition coefficient was *λ*=0.9, the labelling efficiency was *ε*=0.8×0.75 (combined efficiency of labelling and suppression), the T1 of blood was T1_B_ = 1.6 at 3 T, the saturation recovery time for proton density–weighted image was T_SAT_ = 2.0 s, the correction for saturation recovery on proton density–weighted image was T1_GM_ = 1.2 s, and the number of excitations (nex) = 2. ASLdiff was the ASL difference image and PDref was the proton density–weighted reference image.

### Evaluation of SUVR/CBF abnormality asymmetry index

We calculated an asymmetry index (AI) to detect left–right asymmetry in the FDG PET SUVR and ASL-MRI CBF data based on the following equation 1 [35]:

1 $$ \mathrm{AI}=\left(\mathrm{H}-\mathrm{L}\right)/\mathrm{H}\times 100\% $$

where H (L) is the value on the contralateral (ipsilateral) side. The normalization of the images allowed the identification of left–right asymmetries in the cerebral hemispheres. Voxel-wise AIs for both the SUVR and CBF maps of each patient were then calculated. Abnormal asymmetry levels were defined as those greater than 10% [36].

To assess the changes in CBF and SUVR after surgery bypass, the parameters ΔCBF and ΔSUVR, which are defined in Eqs. 2 and 3, were calculated as follows [25]:

2 $$ \Delta  \mathrm{CBF}=\left(\mathrm{CBF}\left(\mathrm{postprocedural}\right)/\mathrm{CBF}\left(\mathrm{preprocedural}\right)-1\right)\times 100\% $$

3 $$ \Delta  \mathrm{SUVR}=\left(\mathrm{SUVR}\left(\mathrm{postprocedural}\right)/\mathrm{SUVR}\left(\mathrm{preprocedural}\right)-1\right)\times 100\% $$

### Statistical analysis

All averaged data are expressed as mean ± standard deviation (SD). Paired *t* tests were used to compare the differences in SUVR and CBF between the patients and between measurements obtained before and after the surgical intervention. All tests were considered significant at the *P* < 0.05 level. Spearman’s rank correlation was also used to correlate CBF and glucose metabolism.

## Results

**Fig. 1:**
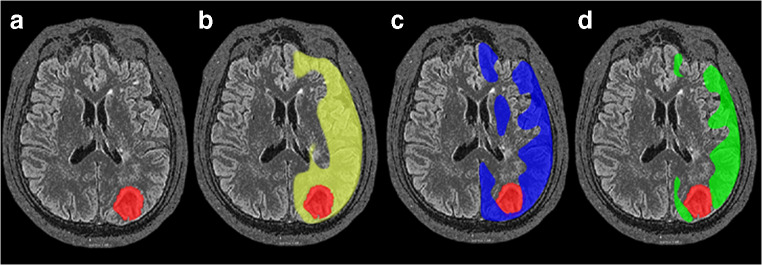
Diagrams showing ROIs in a 3-dimentional stereotaxic ROI template. Red areas indicate the infarct zone (**a**). Yellow and blue regions indicate CBF-decreased regions (**b**) and SUVR-decreased regions (**c**), respectively. The common regions where CBF and SUVR both decreased are showed in green (**d**)

**Fig. 2:**
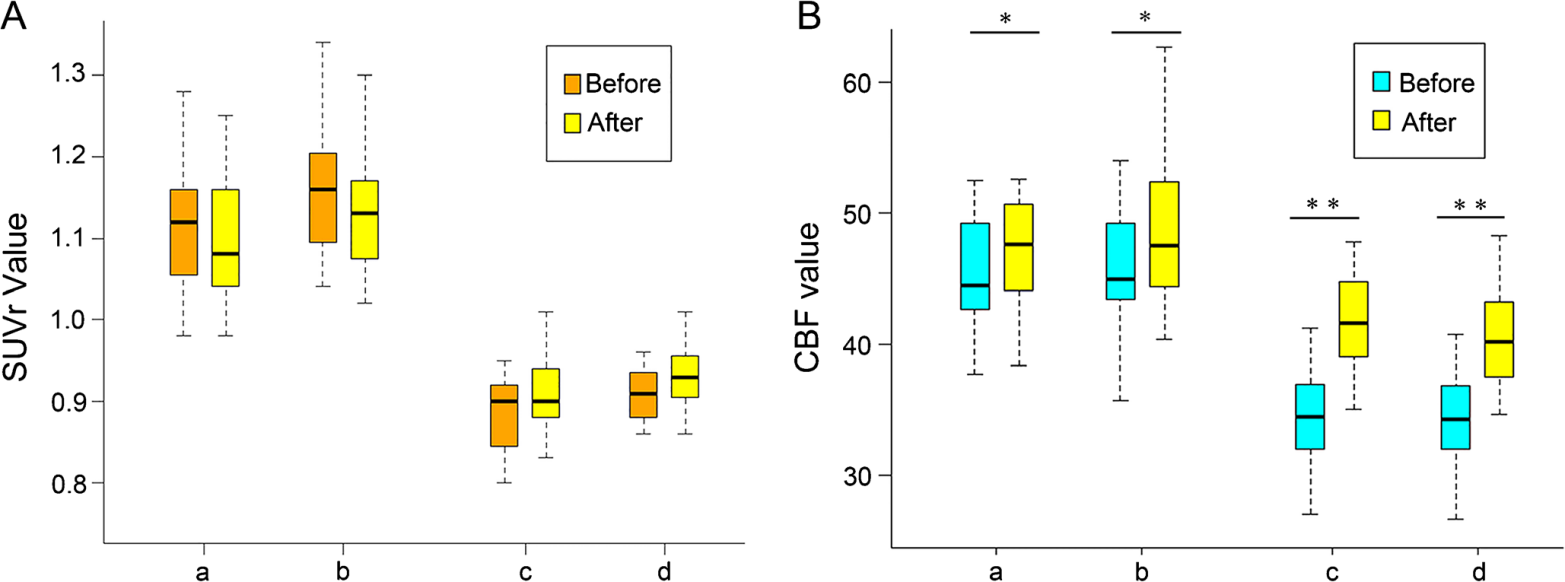
Comparison of preoperative and postoperative SUVR values. (A) Neither the contralateral hemisphere (a, decreased metabolism region; b, synchronous region) nor the ipsilateral hemisphere (c, decreased metabolism region; d, synchronous region) showed significant improvement. (B) Comparison of preoperative and postoperative CBF values. Both the contralateral hemisphere (a, blood flow-decreased region; b, synchronous region) and the ipsilateral hemisphere (c, blood flow–decreased region; d, synchronous region) showed significant improvement. *P < 0.05, **P < 0.01, before vs after surgery

**Fig. 3:**
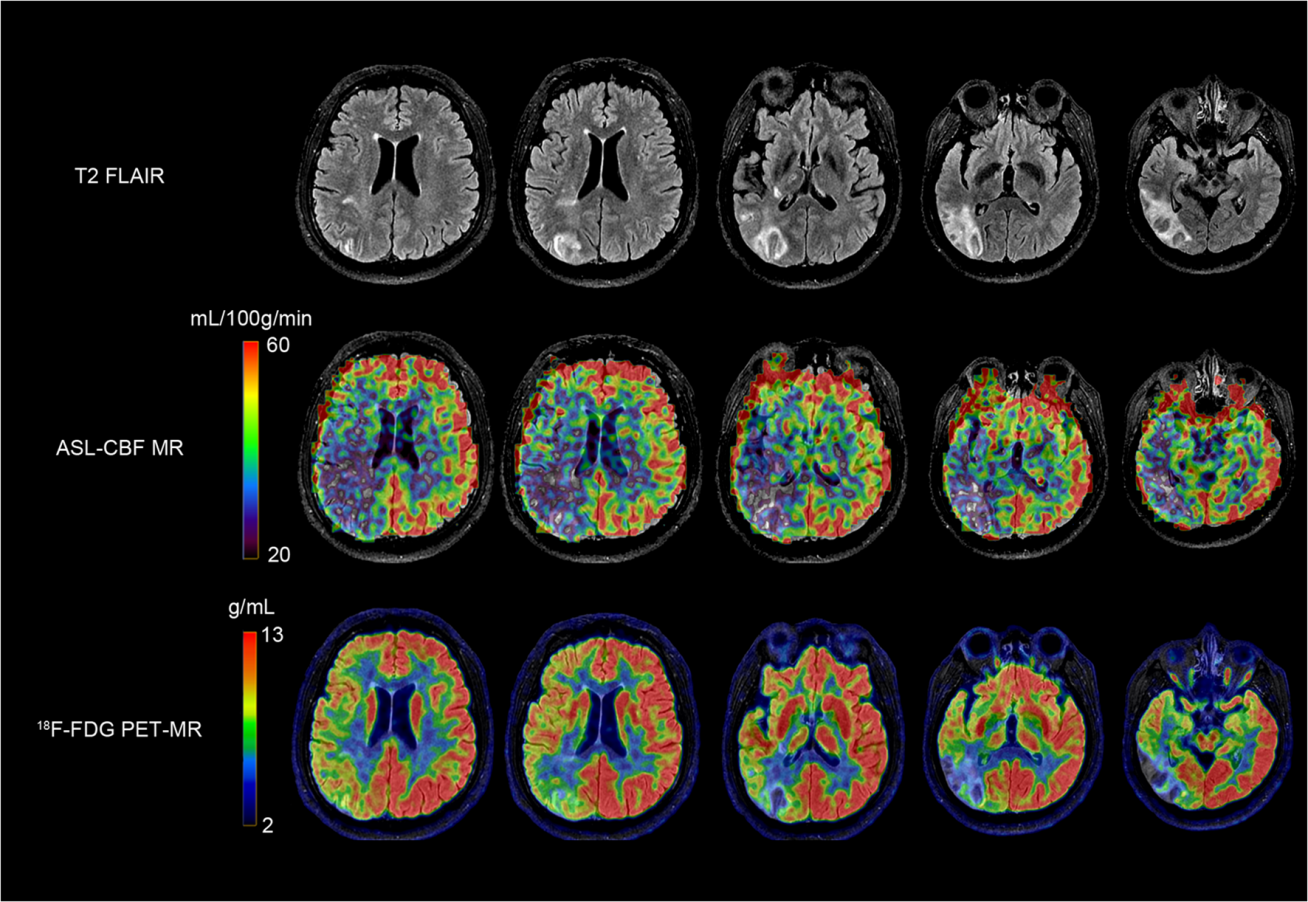
A 43-year-old man with symptomatic occlusion of the right ICA. T2 FLAIR images acquired at baseline showed infarction in the right occipital and temporal lobe. Both ASL-CBF and ^18^F-FDG PET showed that CBF and ^18^F-FDG uptake (right) were reduced relative to finding in the contralateral hemisphere, with CBF AI and SUVR AI values of 37.55% and 30.73%, respectively

Angiographic studies demonstrated that ICA occlusion occurred in 10 patients, ICA stenosis in 1 patient, MCA stenosis in 1 patient, and MCA occlusion in 3 patients. A 3-dimensional region of interest (ROI) template was used to automatically place both ipsilateral and contralateral hemispheres (Fig. 1). Before the operation, ASL and PET were used to measure CBF and SUVR values, respectively, in 15 patients with ICA/MCA stenosis or occlusion. The mean value ± SD of the CBF-decreased regions on the affected side, not including the infarction zone, was 34.38 ± 3.76 mL·100 g^−1^ min^−1^. The mean value ± SD of the SUVR in the decreased regions on the affected side, not including the infarction area, was 0.88 ± 0.05. The synchronous region, defined as where both CBF and SUVR decreased (shown in Fig. 1d), showed that the CBF and SUVR values were 33.78 ± 4.00 (mL·100 g^−1^ min^−1^) and 0.90 ± 0.05, respectively. Figure 2 A and B show that CBF and SUVR were significantly lower in the side ipsilateral to the ICA/MCA stenosis or occlusion than in the contralateral side (*P* < 0.01). Figure 3 provides an example of data obtained in a patient before surgery.

**Fig. 4:**
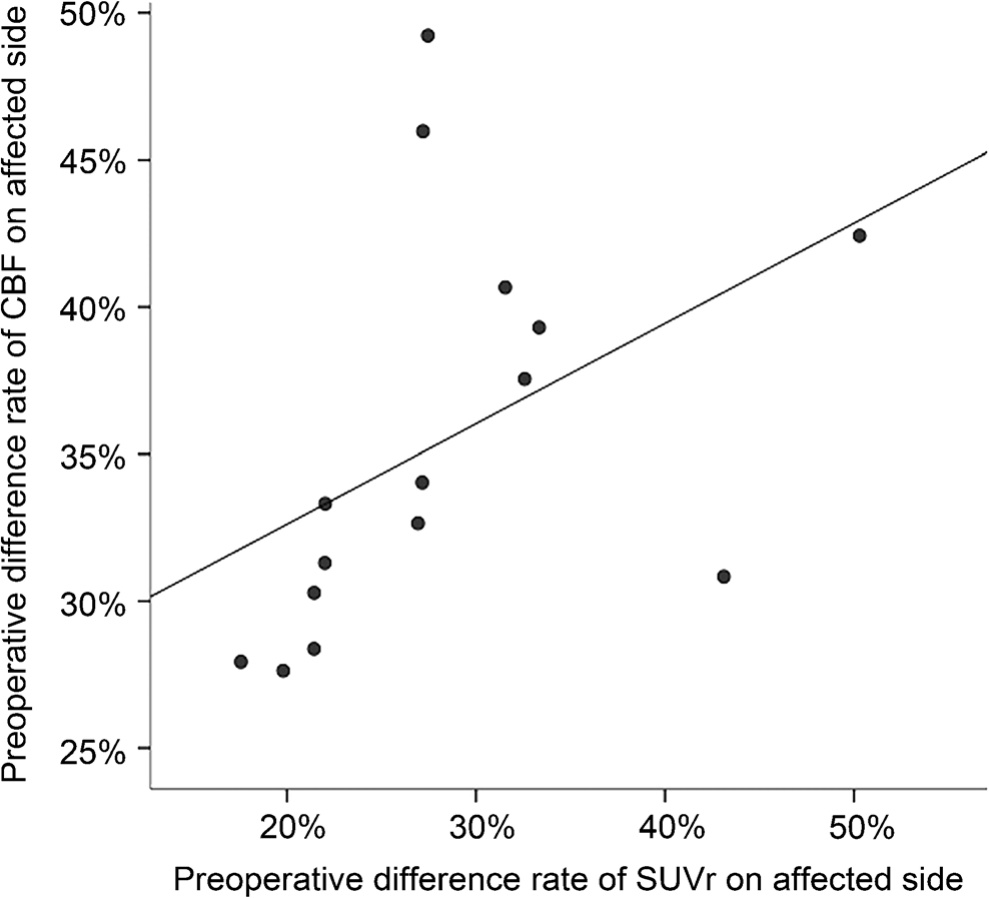
Correlation across the 15 patients, in the same regions of interest between preoperative and postoperative PET and ASL AI values (*r* = 0.729, *p* < 0.01)

A significant correlation was observed between the asymmetry index of preoperative CBF and preoperative SUVR values in 15 patients (*r* = 0.729, *P* < 0.01; Fig. 4), but there was no significant correlation between CBF and glucose uptake measurements obtained on the affected side (*P* > 0.05).

Figure 2 A and B also show the changes that occurred in CBF and SUVR values after the operation. The mean preoperative values ± SD of the regions with lower CBF on the zone on the affected side (without the infarction area) before the surgery was 41.59 ± 3.83 (mL·100 g^−1^ min^−1^). The mean preoperative values ± SD of the regions with lower SUVR on the affected side (without the infarction area) was 0.91 ± 0.05. The corresponding values in the synchronous region including the lower CBF and lower SUVR areas on the ipsilateral side were 40.84 ± 4.08 (mL·100 g^−1^ min^−1^) and 0.93 ± 0.04, respectively (Table 2).

**Table 2:** Pre- and postprocedural CBF and SUVR values obtained in the ipsilateral and contralateral sides

	ROI	SUVR				CBF (mL·100 g^−1^ min^−1^)			
		Preprocedural	Postprocedural	Change	*P* value	Preprocedural	Postprocedural	Change	*P* value
Ipsilateral (surgery side)	Decreased metabolism region	0.88 ± 0.05	0.91 ± 0.05	2.89 ± 5.35%	0.057				
	Blood flow decreased region					34.41 ± 3.76	41.59 ± 3.83	21.73 ± 12.03%	*P* < 0.01
	Synchronous region	0.90 ± 0.05	0.93 ± 0.04	3.29 ± 6.17%	0.059	33.78 ± 4.00	40.84 ± 4.08	21.75 ± 12.43%	*P* < 0.01
Contralateral (non-surgery side)	Decreased metabolism region	1.12 ± 0.08	1.10 ± 0.08	− 1.65 ± 4.59%	0.177				
	Blood flow decreased region					45.21 ± 4.61	47.84 ± 5.33	6.18 ± 9.86%	0.035
	Synchronous region	1.15 ± 0.08	1.13 ± 0.09	− 1.82 ± 4.84%	0.16	45.64 ± 4.93	48.84 ± 5.85	7.45 ± 11.07%	0.026

**Fig. 5:**
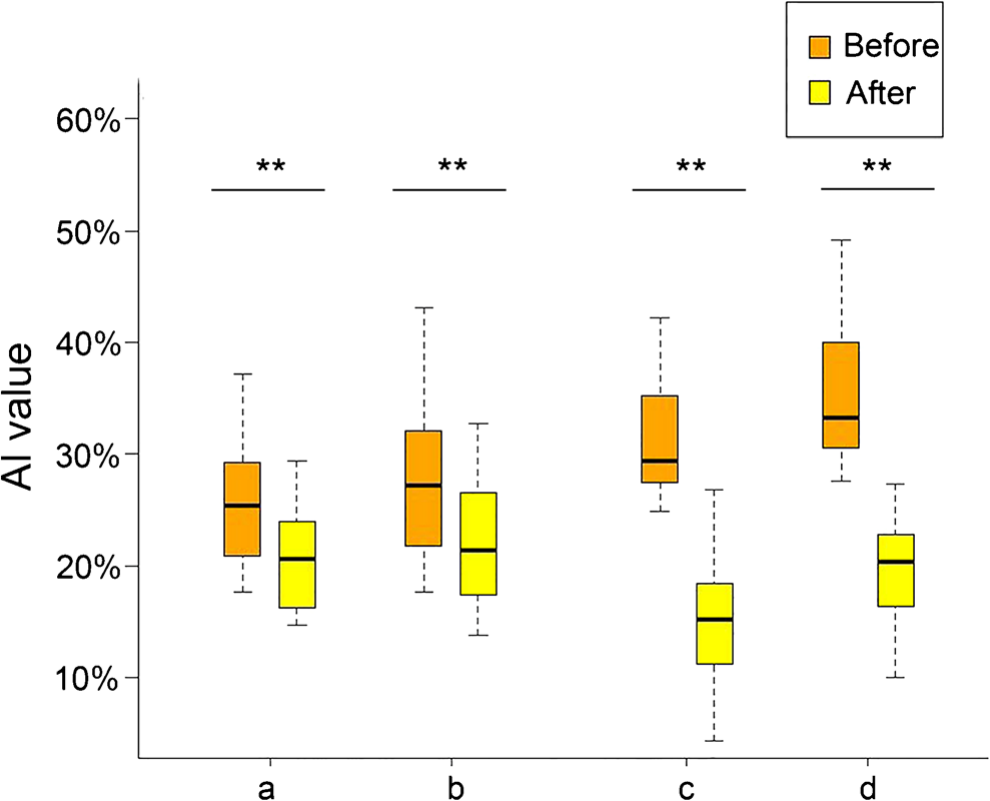
Comparison of preoperative and postoperative AI values. SUVR AI values in decreased metabolism regions (a), and the synchronous zone (b) and CBF AI values in the zone with decreased blood flow (c) and the synchronous zone (d) were lower postoperatively than preoperatively. **P < 0.01 after vs before surgery

**Fig. 6:**
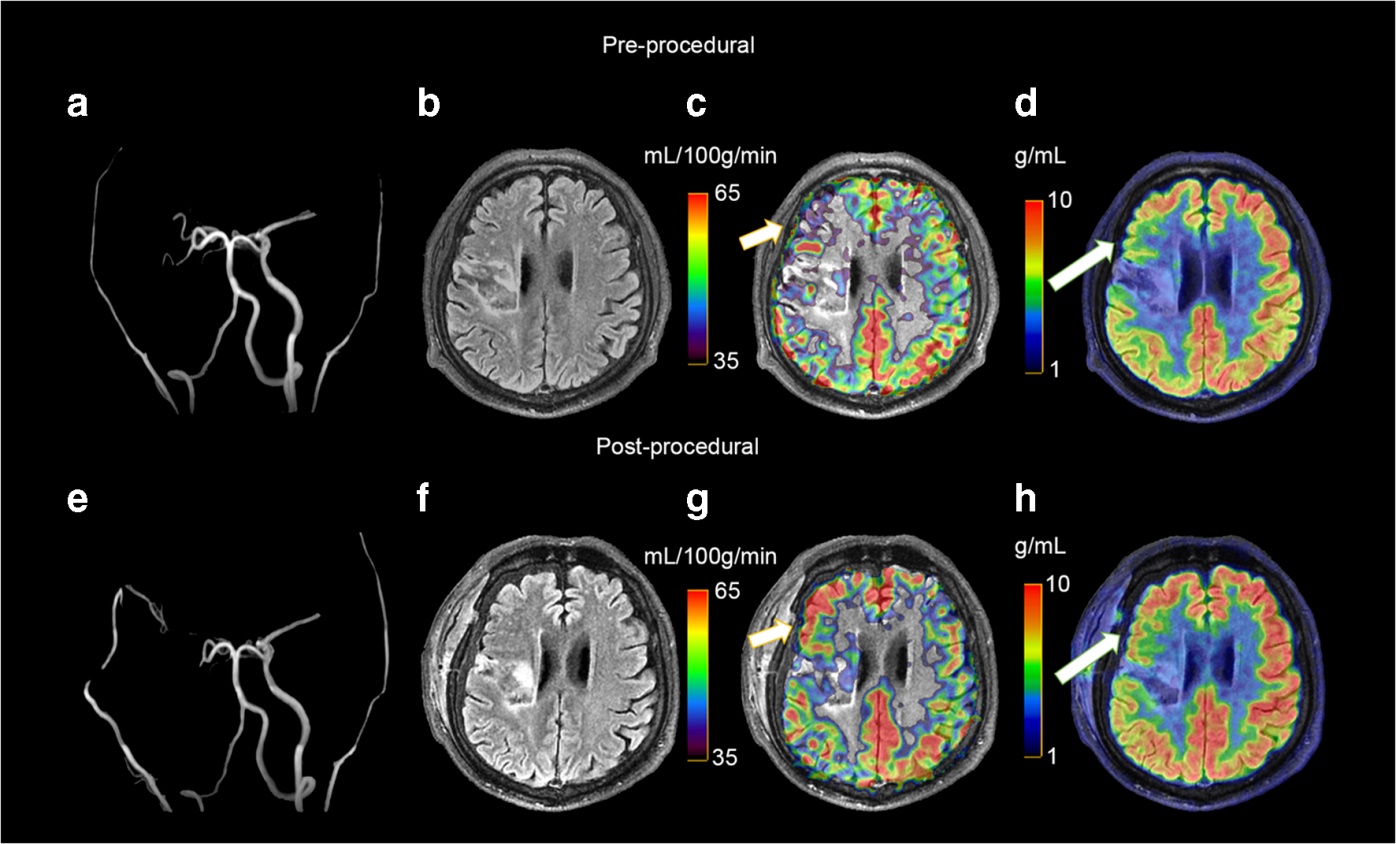
Representative preprocedural and postprocedural images obtained in a 51-year-old man with symptomatic occlusion of the right ICA who underwent bypass surgery. Preoperative MRA (a) showed severe ICA occlusion, and postoperative MRA (e) showed an end-to-side anastomosis between the STA and MCA. MRA T2 FLAIR images (b and f) showed infarction in the left radial crown and corpus callosum. Postprocedural fused CBF/MR image (g) showed improvement compared to the preprocedural image (c) on the affected side (short arrow). Postprocedural fused ^18^F-FDG PET/MR image (h) showed improvement compared to the preprocedural image (d) on the ipsilateral side (long arrows)

CBF was significantly higher postprocedure than preprocedure for the same regions (*P* < 0.05 in both), but the postprocedural SUVR was not significantly higher than the preprocedural SUVR (*P* > 0.05). However, Fig. 5 shows that the postprocedural Al values obtained for of CBF and SUVR were significantly lower than the corresponding preprocedural values (*P* < 0.05). An example of the preoperative PET/MR map of obtained in an STA-MCA bypass patient is shown in Fig. 6.

## Discussion

This study highlights the potential of PET/MR as a noninvasive imaging tool for investigating haemodynamic and metabolic changes between before and after bypass surgery. Our results show that a combination of ASL and ^18^F-FDG PET can be used to optimally evaluate MR-negative regions based on structural images in unilateral ICA or MCA occlusive or stenosis disease.

Similar to ^15^O-H_2_O PET, ASL sequences have shown great promise for the assessment of many brain disorders. CBF value plays an important role in diagnosis and treatment selection in patients with cerebrovascular disease. Amin-Hanjani et al. showed that regional hypoperfusion is an important predictive factor for stroke risk [13]. A previous study demonstrated that chronic hypoperfusion is indicated by decreased CBF (< 35.0 mL/100 g/min) in hemispheres with arterial disease [14]. Another study of 155 patients with chronic ischaemic cerebrovascular disease found that perfusion was 38.1 ± 10.9 (mL·100 g^−1^ min^−1^) on the affected side and that bypass surgery obviously improved CBF values [37]. In our study, the mean value ± SD of the CBF on the affected side was 34.38 ± 3.76 (mL·100 g^−1^ min^−1^) before the operation, comparable to previous studies [14, 37–39]. Bypass surgery aims to increase blood flow to the affected hemisphere and reduce the risk of ensuing neuronal damage in patients. Most studies have documented the clinical benefits and hemodynamic improvements observed after revascularization surgery. These studies have shown that perfusion is greatly improved after treatment [9, 10, 25, 37]. Our results demonstrated that there was significant improvement between the preprocedural and postprocedural CBF values and that the CBF value for the contralateral side also increased after surgery. Soinne et al. investigated CBF changes in patients who underwent carotid endarterectomy and suggested that this procedure increases CBF values on both the side of the surgical procedure and the contralateral side [40]. Analogous findings have been presented in other studies [25]. This phenomenon may be due to the postoperative increase in blood flow on the affected side, which causes a compensatory increase in the contralateral blood flow.

The brain is a highly metabolically active organ that relies on glucose as its energy source. ^18^F-FDG is a tracer of regional brain glucose metabolism. Although it is a widely used radiopharmaceutical agent in the metabolic evaluation of oncology and cardiology patients, it may also offer valuable insights into cerebral glucose metabolism during cerebral ischaemia. In fact, many studies have evaluated the potential of ^18^F-FDG in cerebral ischaemia [17–19, 41]. Fukumoto et al. carried out a serial PET study in which ^18^F-FDG was used to investigate changes in glucose metabolism before and at 1, 3, 7, and 14 days after acute stroke in an animal model and found that glucose metabolism was comparable between the peri-ischaemic areas and normal brain regions on days 1 and 3 but significantly higher in the later on days 7 and 14. Furthermore, some studies have observed that ^18^F-FDG uptake is higher in peri-ischemic regions, similar to findings in animal studies [19]. However, all the studies we read explored acute stroke, and little research has focused on chronic stroke. Nagasawa H et al. found that there were significant decreases in cerebral glucose metabolic rate (CMRGlu) in areas in which no lesions had been detected based on using ^18^F-FDG PET in MRI or CT scan in 7 patients with chronic ischemic cerebrovascular disease [20]. Yu Z et al. previously reported seven patients who demonstrated improved glucose metabolism on ^18^F-FDG PET after bypass surgery [28]. In the present study, we also focused on the changes that occurred in glucose metabolism between before and after bypass surgery in chronic ischaemic cerebrovascular disease. Our results show there was no significant increase in SUVR between preprocedural and procedural measurements. However, SUVR AI values were significantly lower after surgery than before surgery. This change in SUVR and AI values might indicate that surgery plays a pivotal role in preventing irreversible ischaemic damage in steno-occlusive artery disease. However, a follow-up duration of 7 days after bypass surgery might be too short. CBF values increased significantly during these 7 days. Hence, the promotion of glucose metabolism seems to involve a slower process and requires a longer follow-up.

Cha et al. compared resting state cerebral perfusion evaluated using ASL and glucose metabolism evaluated using ^18^F-FDG PET in 20 normal volunteers and found a good overall correlation between perfusion and glucose metabolism [42]. Yu Z et al. found that bypass surgery changed CBF and FDG uptake of chronic ischaemic cerebrovascular disease [28]. However, they did not obtain these data simultaneously. The optimal way to avoid functional and physiological variations in a multimodal study is to simultaneously acquire data using different modalities. The advantage of our study is that we established a method of measuring cerebral perfusion and glucose metabolism while also assessing collateral flow patterns with a single modality in a single session. Our data showed that although there was an overall good correlation between the AI of CBF measured by ASL and the AI of SUVR measured by ^18^F-FDG PET, both were significantly lower after surgery. It is clear that the changes that occurred in CBF and SUVR were more dramatic in the synchronous region than in either the CBF-decreased areas alone or the SUVR-decreased areas alone. Ischaemic cerebrovascular disease is a fatal condition that arises due to insufficient blood flow and metabolic impairment to damages brain cells [43, 44]. Theoretically, vascular stenosis or occlusion immediately affected perfusion and causes metabolic changes in the area of the vascular distribution. However, our results show that the area with blood flow reduction and the area with metabolic reduction were not completely overlapping. Some researchers have suggested that this blood flow metabolism mismatch is due to a compensatory or inflammatory response [45, 46]. In the case of FDG PET measurements, test/retest studies have shown that there is variability in their ranges [47, 48]. However, we chose to explore the difference between the SUVR of the ipsilateral region and the SUVR of the contralateral region to directly evaluate the metabolism of glucose. Thus, rCMRglc can be regarded as a stable value in our studies and showed improvements were achieved in some areas. In the common area, both CBF and SUVR decreased and were negative in a structural image. Based on stroke mechanisms, I propose that the common area might have a higher risk of infarction. The effectiveness of treatment is important in patients with chronic ischaemic cerebrovascular disease. However, its exact impact needs further research

This study also has some limitations. First, the follow-up time was short: Since the natural temporal course of brain FDG metabolism and changes in CBF in carotid artery stenosis or occlusion are unknown, the optimal timing remains unclear. Hence, the study follow-up times should be extended in both number and duration. Second, we should enlarge the study sample size. Third, our study shows that the postlabelling delay (PLD) is the most important parameter when measuring CBF; hence, multiple PLDs should be chosen. While SUVR is a relative parameter when measuring glucose metabolism, absolute CMRGlu could be measured by dynamic ^18^F-FDG PET using image-derived input functions (IDIF) in an integrated PET/ MRI system. Finally, a cognitive score could be used to systematically investigate cognitive differences among these patients.

## Conclusion

The present study demonstrates that combining CBF measured by ASL and metabolism assessed by ^18^F-FDG using hybrid PET/MR allows the simultaneous analyse of changes in cerebral haemodynamic patterns and metabolism both before and after STA–MCA bypass surgery and may therefore represent an essential tool for the evaluation of critical haemodynamic and metabolic status in patients with symptomatic unilateral ischaemic cerebrovascular disease.
